# Risk of Newly Diagnosed Psychotic Symptoms in Youth Receiving Medications for Attention-Deficit/Hyperactivity Disorder

**DOI:** 10.1016/j.jaacop.2024.01.003

**Published:** 2024-02-05

**Authors:** Rana Elmaghraby, Andrew Pines, Jennifer R. Geske, Brandon J. Coombes, Jonathan G. Leung, Paul E. Croarkin, Matej Markota, William V. Bobo

**Affiliations:** a Cincinnati Children’s Hospital Medical Center. Cincinnati, Ohio; b Bringham and Women’s Hospital, Harvard Medical School, Boston, Massachusetts; c Mayo Clinic, Rochester, Minnesota; d Mayo Clinic, Florida, Jacksonville, Florida

**Keywords:** attention-deficit/hyperactivity disorder, nonstimulants, psychosis, stimulants

## Abstract

**Objective:**

Epidemiological studies suggest that patients with attention-deficit/hyperactivity disorder (ADHD) treated with amphetamines have an increased risk of newly diagnosed psychosis. This risk in youth is poorly understood. This investigation studied the potential risk of newly diagnosed psychotic symptoms associated with exposure to 4 classes of ADHD medications.

**Method:**

This retrospective study used a medical records–linkage system from a cohort of youth (age 6-18 years) with diagnosed ADHD who were prescribed amphetamines, methylphenidate, atomoxetine, or α-2 agonists. Cohort members with any diagnosis of psychosis before their first ADHD medication were excluded. The primary outcome was newly diagnosed psychotic symptoms. The risk for psychotic symptoms for each medication (vs the remaining medication classes combined) was estimated using a multivariable time-varying covariate Cox proportional hazard regression model that adjusted for sex and age at ADHD diagnosis.

**Results:**

Of 5,171 youth (68.6% male), 134 (2.6 %) had newly diagnosed psychotic symptoms. Exposure to amphetamine (vs amphetamine nonexposure, hazard ratio 1.41, 95% CI 1.15-2.26) and atomoxetine (vs atomoxetine nonexposure, hazard ratio 2.01, 95% CI 1.38-2.92) was associated with increased risk of newly diagnosed psychotic symptoms. Secondary analysis showed that the frequency of newly diagnosed psychotic symptoms was higher with atomoxetine/stimulant lifetime combination therapy (12.5% with amphetamines, 7.7% with methylphenidate) than atomoxetine monotherapy (1.2%).

**Conclusion:**

Risk of newly diagnosed psychotic symptoms was low. These results suggest that cumulative exposure to amphetamines or atomoxetine/stimulant lifetime combination therapy may be associated with an increased risk of newly diagnosed psychotic symptoms in youth with ADHD.

**Diversity & Inclusion Statement:**

We worked to ensure race, ethnic, and/or other types of diversity in the recruitment of human participants. We worked to ensure that the study questionnaires were prepared in an inclusive way. We worked to ensure sex and gender balance in the recruitment of human participants. One or more of the authors of this paper self-identifies as a member of one or more historically underrepresented racial and/or ethnic groups in science. One or more of the authors of this paper received support from a program designed to increase minority representation in science. We actively worked to promote sex and gender balance in our author group. We actively worked to promote inclusion of historically underrepresented racial and/or ethnic groups in science in our author group. While citing references scientifically relevant for this work, we also actively worked to promote sex and gender balance in our reference list. While citing references scientifically relevant for this work, we also actively worked to promote inclusion of historically underrepresented racial and/or ethnic groups in science in our reference list. The author list of this paper includes contributors from the location and/or community where the research was conducted who participated in the data collection, design, analysis, and/or interpretation of the work.

## Plain language summary

This retrospective study, using the Rochester Epidemiology Project, looked at the risk of developing newly diagnosed psychotic symptoms in youth (n = 5,171 between 6- to 18-years-old) with attention-deficit/hyperactivity disorder (ADHD) treated with 4 different classes of US FDA approved medications. The authors found that the overall absolute risk of psychosis was low. The risk of newly diagnosed psychotic symptoms was higher in youth treated with amphetamines and atomoxetine. In addition, this risk was observed when atomoxetine was combined with methylphenidate, amphetamine, or both. Authors suggest that this association may be related to attentional difficulties presenting as a prodromal symptom of psychosis or related to polypharmacy in youth with treatment-resistant ADHD.

Attention-deficit/hyperactivity disorder (ADHD) is one of the most common psychiatric disorders in youth.^1^ Approved medications for the treatment of ADHD in youth in the United States include stimulants (amphetamines and methylphenidate) and nonstimulants (α-2 agonists and atomoxetine). According to current guidelines, stimulants (amphetamines and methylphenidate) are first-line treatments for the management of ADHD.^2^ In preschool-age patients with ADHD, amphetamines are the only US Food and Drug Administration (FDA)–approved medication, although guidelines suggest that methylphenidate rather than amphetamines may be helpful if behavioral interventions prove insufficient.^2^, ^3^, ^4^ The two other FDA-approved ADHD pharmacotherapeutic agents are α-2 agonists and atomoxetine. Atomoxetine is used when there is concern for illicit substance use because of low potential of abuse, and α-2 agonists are used when there is poor response to stimulants, intolerable side effects to stimulants, and/or significant coexisting conditions (eg, tics).^2^^,^^5^

Methylphenidate and amphetamines both block the dopamine transporter and thereby increase levels of extracellular dopamine in the striatum.^5^, ^6^, ^7^, ^8^ Amphetamines are 4 times more likely to increase dopamine availability in the synaptic cleft compared with methylphenidate.^5^ Amphetamine results in more presynaptic dopaminergic release via increased extracellular dopamine through 2 mechanisms not exhibited by methylphenidate: inhibition of the vesicular transporter and reversal of the dopamine transporter.^5^ The α-2 agonists, such as clonidine and guanfacine, stimulate postsynaptic receptors and result in increase in norepinephrine signaling.^9^ Atomoxetine blocks norepinephrine and dopamine reuptake.^9^

The pathophysiology of psychosis in patients with schizophrenia or bipolar disorder is thought to involve an increased dopamine tone in the mesolimbic pathway.^10^, ^11^, ^12^, ^13^ The similarity in physiology between psychosis and stimulant use has led investigators to question if the use of amphetamines and methylphenidate can increase the risk for psychosis.

Several studies have raised concerns for an association between risk for new-onset psychosis (defined as new diagnosis code of psychosis and a prescription of an antipsychotic) and stimulants treatment for ADHD.^14^, ^15^, ^16^ One prospective study of 98 stimulant-treated children with ADHD documented the occurrence of incident psychotic symptoms in 6 (6.1%) participants over 9 to 12 months of follow-up.^17^ A more recent study reported significantly higher risk of newly diagnosed psychotic illness in youth with ADHD who were treated with amphetamines than youth with ADHD who were treated with methylphenidate (hazard ratio [HR] with amphetamine use 1.65, 95% confidence interval 1.31-2.09).^15^ Bramness and Rognli^16^ reported that having a family history of psychosis is a potential risk factor for acquiring psychotic symptoms. Most studies did not report on family history of psychosis or prodromal symptoms as potential risk factors, and this remains an unanswered question in the literature. Most studies included a cohort of adults making prodromal symptoms less concerning as they would be in a cohort of adolescents and young adults.

Few studies have examined the risk of incident psychosis with nonstimulant ADHD treatments. In a pooled analysis of postmarketing surveillance data, 14 of the 49 studies documented an increased risk of symptoms of psychosis and mania among atomoxetine-treated youth with ADHD compared with placebo (4 psychosis/mania events in 487.5 person years).^14^ This pooled analysis noted that no risk factors were identified that could account for the cases of psychotic or manic symptoms. In a separate pooled analysis of multiple short-term placebo-controlled studies, atomoxetine was associated with an increased risk of symptoms of psychosis and mania in atomoxetine-treated children with ADHD, age 6 to 18 years, compared with placebo (0 psychotic reactions among 1,056 patients) (data from Eli Lilly and Company, Indianapolis, Indiana; https://pi.lilly.com/ca/strattera-ca-pm.pdf). Similar to other studies, no risk factors, such as family history of psychosis or prodromal symptoms, were reported.^14^^,^^15^^,^^18^^,^^19^

Concerns regarding the associations between atomoxetine and new-onset psychotic and/or manic symptoms prompted an FDA-mandated change in drug labeling to include warnings about these potential risks in 2007.^1^ In 2022, the FDA provided additional guidance, recommending discontinuation of atomoxetine in patients who develop treatment-emergent psychotic symptoms^14^ (https://www.accessdata.fda.gov/drugsatfda_docs/label/2009/021411s029s030lbl.pdf). The associations of other types of nonstimulants and newly diagnosed psychotic symptoms are unknown. To our knowledge, no studies have compared the risk of new-onset psychotic symptoms associated with stimulant and nonstimulant medication classes in a single cohort of youth with ADHD. Furthermore, there is limited understanding of whether a possible association between atomoxetine and new-onset psychotic symptoms reflects an interaction with co-prescribed stimulants, which is an important clinical consideration given the increasing rates of ADHD polypharmacy among patients age 2 to 24 years.^20^ Finally, little is known about the risk of new-onset psychotic symptoms beyond 60 days of initiating pharmacotherapy for ADHD. The goal of this study was to test the associations between exposure to 4 main classes of FDA-approved medications (amphetamines, methylphenidate, selective norepinephrine reuptake inhibitor [atomoxetine], and α-2 agonists) and new-onset psychotic symptoms in youth with a diagnosis of ADHD. By identifying an association, we hypothesized that a subgroup of youth with ADHD treated with medications are at heightened risk of newly diagnosed psychotic symptoms and that a combination of medications could increase that risk as well.

## Method

### Data Source

The Rochester Epidemiology Project (REP) was used to identify a geographically defined cohort of youth residing in 9 adjacent Minnesota counties. The REP is a comprehensive medical records–linkage system for virtually all persons residing in Olmsted County and its adjacent counties in Minnesota and Wisconsin that has existed for more than 50 years.^21^ The REP provides a unique infrastructure for research, and the information in the electronic REP files captured nearly the entire population of the Minnesota counties included in the study, as compared to the US census estimates.^5^

Cohort members received their first ADHD treatment with amphetamines, methylphenidate, atomoxetine, or α-2 agonist between January 1, 1998, and December 31, 2018, and were 6 to 18 years of age at the time of treatment. Data on prescribed medications were obtained from electronic REP files that contained structured information on prescriptions including drug name, schedule of administration, prescription dates, amount to be dispensed, and number of refills. Data on diagnoses were obtained electronically from REP indices in the form of *ICD-9* and *ICD-10* diagnosis codes, with their corresponding dates. The REP did not include data on family history of psychosis. This study received approval from local Institutional Review Boards from the Mayo Clinic and Olmsted Medical Center in Rochester, Minnesota.

### Study Cohort

**Figure 1:**
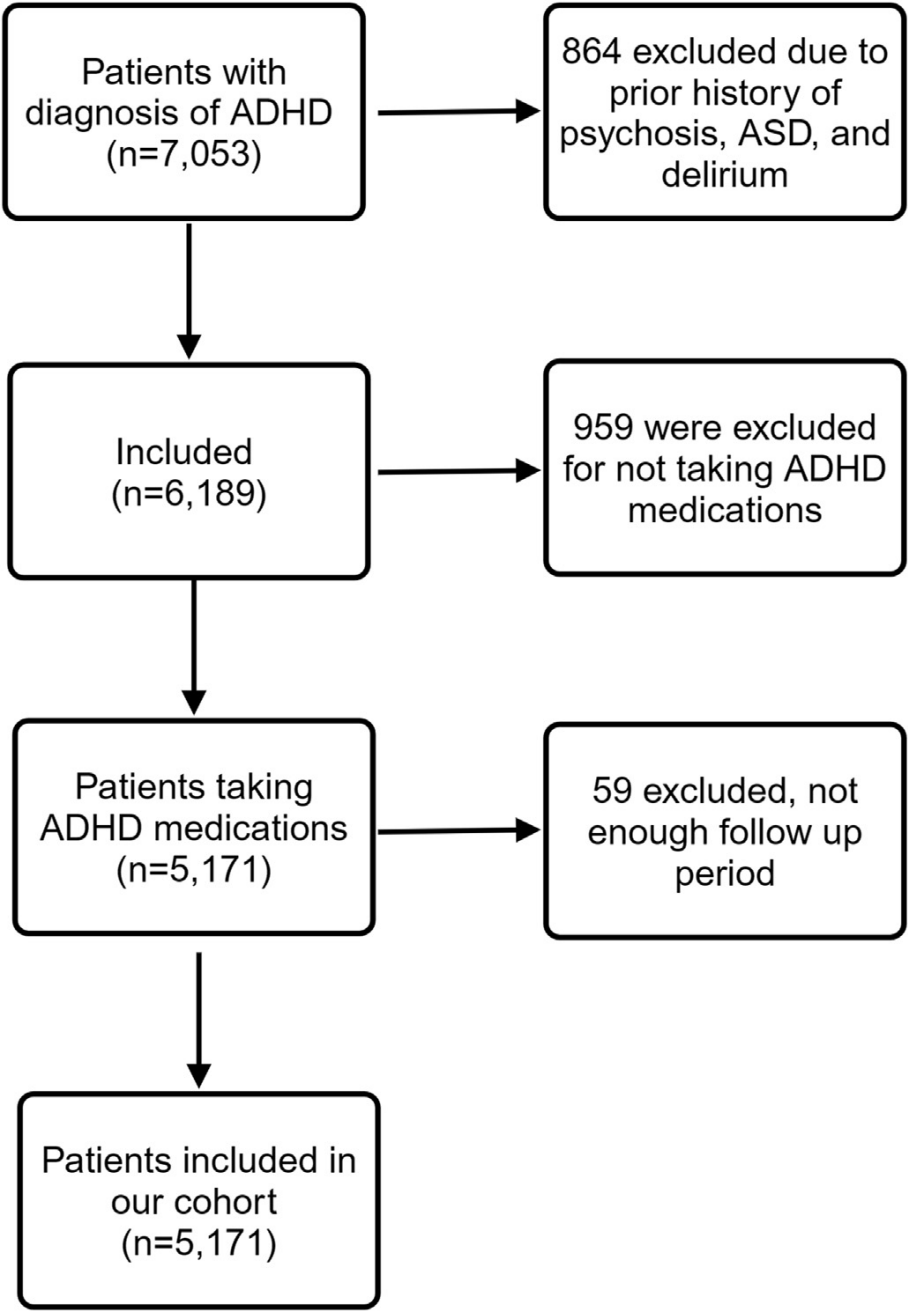
Flow Diagram ***Note:** ADHD = attention-deficit/hyperactivity disorder; ASD = autism spectrum disorder.*

Cohort members with no parental research authorizations on file were excluded, which was less than 4% of the overall population in the Minnesota counties covered by the birth cohort.^21^ Cohort members were excluded if there was evidence of psychotic or mood disorders before receiving their first qualifying medication prescription (*ICD-9* codes 292.1x, 292.2, 292.81, 295x, and 296X and *ICD-10* equivalents) and/or had a lifetime diagnosis of neurodevelopmental disorders (*ICD-9* codes 299x and 317-319x and *ICD-10* equivalents). To optimize diagnostic accuracy, we also excluded youth who had received a diagnosis of delirium (*ICD-9* codes 292.81, 293.0, 293.1, 293.81, and 293.82 and *ICD-10* equivalents), which can mimic psychosis. Cohort members were excluded if they were ever prescribed antipsychotics, mood stabilizers, or ADHD medications before receiving their first qualifying medication prescription. To minimize misclassifying substance-induced psychosis as newly diagnosed psychosis, we excluded cohort members who had any previously diagnosed substance use disorder (based on *ICD* codes), who had a positive urine toxicology, or who had ever received oral glucocorticoid or steroid-based medications (Figure 1). From the REP electronic indices, we collected data on sex assigned at birth, age at first ADHD diagnosis, and self-identified race.

### Exposure

The main exposure was the cumulative time since starting a study medication, which included stimulants (amphetamines and methylphenidate) and nonstimulants (atomoxetine and α-2 agonists). Amphetamines included amphetamine/dextroamphetamine, lisdexamfetamine, mixed amphetamine salts, and dextroamphetamine. Methylphenidate included methylphenidate or dexmethylphenidate. Immediate- and controlled-/extended-release formulations were included for all drug classes. The α-2 agonists included clonidine and guanfacine. The exposure window of a study medication started on the date of the first qualifying prescription and ended when the calculated days of medication supply ended (including small gaps, approximately 15-30 days, needed for refills).

### Follow-Up

Follow-up began on the date of the first qualifying study medication prescription. To increase the likelihood of the follow-up period starting on the date of the first medication prescription, we introduced a 1-year washout period that excluded any cohort members who received a stimulant prescription in the year before the first qualifying medication prescription, which was between January 1, 1998, and December 31, 1998. The end of follow-up was defined as the date of a new (first) diagnosis of psychotic symptoms during the study period (see End Points), death, or December 31, 2018, whichever occurred soonest.

### End Points

Newly diagnosed psychotic symptom was defined using *ICD-9* and *ICD-10* codes in the REP electronic indices (*ICD-9* codes 292.1x, 295x, 296.24, 296.34, 296.44, 296.54, and 297x-298x and *ICD-10* equivalents) (Table S1, available online). Our case definition was based on the presence of a single diagnosis code during follow-up.

### Statistical Analysis

Demographic and clinical characteristics were compared between cohort members who developed newly diagnosed psychotic symptoms and those who did not, using χ^2^ tests for categorical variables and t tests for continuous variables. Statistical comparisons focused on the risk of newly diagnosed psychotic symptoms conditioned on study medication exposure using time since first exposure to each medication as a predictor. To test for associations of cumulative time since exposure to each medication with newly diagnosed psychotic symptoms, we used a multivariable time-varying covariate Cox proportional hazard regression model with adjustment for age at diagnosis of ADHD and sex assigned at birth. Self-reported race was not a covariate in the model given that a large majority of the cohort was White. The effect estimates for each medication type were calculated as adjusted HRs for newly diagnosed psychotic symptoms comparing cohort members exposed to a specific medication (eg, amphetamines) with cohort members not exposed to that medication (eg, amphetamine nonexposure). All cohort members were exposed to at least one ADHD medication during the study period. Therefore, the HRs in this study did not include comparisons with nonusers or former users of ADHD medications.

As an exploratory analysis, we estimated the rate of newly diagnosed psychotic symptoms on each medication combination for cases in which 2 or more ADHD medications were being used. These were lifetime combinations of medications. For example, participants could be taking atomoxetine and a stimulant concomitantly or at separate points in time during follow-up. Given the differences in the pharmacodynamic profiles of ADHD medications in this study, we focused on specific medication combinations rather than studying the effects of ADHD combination therapy as a single exposure. However, due to small numbers of exposures, we did not include specific combinations in the multivariable Cox proportional hazard regression models. Instead, unadjusted odds ratios (ORs) were calculated to compare the occurrence of newly diagnosed psychotic symptoms on a specific medication combination compared with all other medication combinations, while acknowledging that each comparison is not independent and could be confounded by other factors. All analyses were conducted using SAS version 9.4 (SAS Institute Inc., Cary, North Carolina).

## Results

### Demographic and Clinical Information

This study comprised 5,171 youths (Figure 1). As shown in Table 1, cohort members had an average of 7.7 years of follow-up, totaling 39,817 person years. Age at diagnosis of ADHD was slightly older for cohort members with newly diagnosed psychotic symptoms compared with those without newly diagnosed psychotic symptoms (10.8 years old vs 10.2 years old, *p* < .05) (Table 1). The study cohort was predominantly White (83.5%) and male (68.6%). There was no significant difference in self-reported race between cohort members with and without newly diagnosed psychotic symptoms (Table 1). After adjusting for age at diagnosis of ADHD, male sex was associated with increased risk of newly diagnosed psychotic symptoms (HR 1.9, 95% CI 1.3-2.7, *p* = .0009). The most frequently prescribed study drug among cohort members was methylphenidate (79%), followed by amphetamines (40.7%), α-2 agonists (25.1%), and atomoxetine (15.0%) (Table 1).

**Table 1:** Demographic Information

	Total cohort (N = 5,171)		Newly diagnosed psychotic symptoms (n = 134)		No newly diagnosed psychotic symptoms (n = 5,037)		*p*
Age at ADHD diagnosis, y							.0495
	**Mean**	**(SD)**	**Mean**	**(SD)**	**Mean**	**(SD)**	
	10.2	(3.6)	10.8	(3.4)	10.2	(3.6)	
	**Median (range)**		**Median (range)**		**Median (range)**		
	9 (6, 18)		10 (6, 18)		9 (6, 18)		
Sex							0.5544
	**n (%)**		**n (%)**		**n (%)**		
Male	3,545 (68.6)		95 (70.9)		3,450 (68.5)		
Female	1,626 (31.4)		39 (29.1)		1,587 (31.5)		
Follow-up duration, y							<.001
	**Mean**	**(SD)**	**Mean**	**(SD)**	**Mean**	**(SD)**	
	7.7	(4.5)	5.6	(3.5)	7.8	(4.5)	
	**Median (range)**		**Median (range)**		**Median (range)**		
	7.2 (0.1, 16.1)		5.4 (0.4, 14.3)		7.2 (0.1, 16.1)		
Medication duration, days							—
	**Median (range)**		**Median (range)**		**Median (range)**		
Atomoxetine	498 (0, 5,772)		363 (0, 4,205)		751 (0, 5,772)		
Amphetamine	747 (0, 5,772)		213 (0, 4,205)		513 (0, 5,772)		
α-2 agonist	629 (0, 5,772)		303 (0, 2,438)		635 (0, 5,772)		
Methylphenidate	0 (0, 5,772)		114 (0, 4,205)		0 (0, 5,772)		
Medication exposure							<.001
	**n (%)**		**n (%)**		**n (%)**		
Atomoxetine	776 (15.0)		31 (4.0)		103 (2.3)		
Amphetamine	2,106 (40.7)		48 (2.3)		86 (2.8)		
α-2 agonist	1,300 (25.1)		20 (1.5)		114 (2.9)		
Methylphenidate	4084 (79.0)		110 (2.7)		24 (2.2)		
Race							.3256^a^
	**n (%)**		**n (%)**		**n (%)**		
Asian	88 (1.7)		3 (2.2)		85 (1.7)		
Black or African American	258 (5.0)		8 (6.0)		250 (5.0)		
Other^b^	378 (7.4)		5 (3.7)		373 (7.5)		
White	4,292 (83.5)		118 (88.1)		4,174 (83.4)		
Unknown	126 (2.5)		0 (0)		126 (2.5)		

Note: ADHD = attention-deficit/hyperactivity disorder.

a Compares White vs all other racial and ethnic categories combined due to small numbers, excluding cohort members classified as unknown.

b American Indian or Alaskan Native, Native Hawaiian or Other Pacific Islander White.

### Risk of Psychotic Symptoms by Medication Class

As shown in Table 2, exposure to amphetamines was associated with an increased risk of newly diagnosed psychotic symptoms during follow-up (HR 1.6, 95% CI 1.1-2.2) compared with nonexposure to amphetamines in adjusted models. Exposure to atomoxetine was also associated with an increased risk of new-onset psychotic symptoms compared with nonexposure to atomoxetine in adjusted models (HR 2.0, 95% CI 1.3-2.9). Neither exposure to methylphenidate nor exposure to α-2 agonists was associated with an increased risk of newly diagnosed psychotic symptoms during follow-up in adjusted models (Table 2).

**Table 2:** Risk of Newly Diagnosed Psychotic Symptoms According to Study Drug Exposure

Predictor	HR	95% CI		*p*
		Lower bound	Upper bound	
Amphetamine exposure	1.61	1.15	2.26	.006
Amphetamine nonexposure	Reference			
Atomoxetine exposure	2.01	1.38	2.92	.0003
Atomoxetine nonexposure	Reference			
α-2 agonist exposure	0.67	0.42	1.06	.086
α-2 agonist nonexposure	Reference			
Methylphenidate exposure	1.02	0.70	1.49	.91
Methylphenidate nonexposure	Reference			
Age at ADHD diagnosis	1.00	0.95	1.04	.91
Male sex, self-reported	1.90	1.30	2.78	.0009

Note: There were 134 cases of newly diagnosed psychosis. Risk of newly diagnosed psychosis was estimated for each study drug class (amphetamine, methylphenidate, atomoxetine, α-2 agonist) using HRs that were calculated using time varying covariate Cox proportional hazard regression models, adjusted for age at ADHD diagnosis and self-reported sex assigned at birth. For a particular study drug class, the risk of newly diagnosed psychosis was compared against the remaining classes of study drugs (referred to as nonexposure to the drug class under investigation). Age at ADHD diagnosis is a continuous variable, so there is no referent group; the HR interpretation for age at ADHD diagnosis is the HR per year for increase in age. Referent group for male sex is female sex. ADHD = attention-deficit/hyperactivity disorder; HR = hazard ratio.

Additional analyses focused on the risk of newly diagnosed psychotic symptoms associated with atomoxetine combined with amphetamines and/or methylphenidate vs atomoxetine monotherapy. As shown in Table 3, there was an increased risk of newly diagnosed psychotic symptoms in cohort members exposed to atomoxetine combined with amphetamines (12.5%, OR 10.2, 95% CI 3.9-26.4), methylphenidate (7.7%, OR 5.9, 95% CI 2.9-12.2), and both amphetamines and methylphenidate (7.1%, OR 5.5, 95% CI 2.5-11.8) during follow-up compared with those exposed to atomoxetine monotherapy (1.7%).

**TABLE 3:** Medication Lifetime Combinations (Arranged From Most Incidents to Least Incidents of Psychotic Symptoms)

Medication lifetime combinations	No. psychotic symptoms/total	%	OR	(95% CI)
Atomoxetine + amphetamine	6/48	12.50	10.18	(3.92, 26.41)
Atomoxetine + methylphenidate + α-2 agonist	5/53	9.43	7.42	(2.7, 20.44)
Atomoxetine + amphetamine + α-2 agonist	3/34	8.82	6.90	(1.96, 24.26)
Atomoxetine + methylphenidate	12/156	7.69	5.93	(2.88, 12.25)
Atomoxetine + methylphenidate + amphetamine	10/140	7.14	5.48	(2.54, 11.82)
Atomoxetine + methylphenidate + amphetamine + α-2 agonist	5/109	4.59	3.43	(1.27, 9.23)
Atomoxetine + α-2 agonist	1/26	3.85	—	
Atomoxetine	2/119	1.68	1.22	(0.28, 5.24)
Methylphenidate + α-2 agonist	17/397	4.28	3.20	(1.68, 6.06)
Methylphenidate + amphetamine	25/690	3.62	2.68	(1.5, 4.79)
Amphetamine	14/501	2.79	2.05	(1.04, 4.04)
Methylphenidate + amphetamine + α-2 agonist	7/271	2.58	1.89	(0.8, 4.47)
Methylphenidate	42/1,869	2.25	1.64	(0.97, 2.76)
α-2 agonist	2/96	2.08		—
Amphetamine + α-2 agonist	1/90	1.11		—
Total	152/4,599	3.31		

Note: OR represents the comparison of psychosis rate of that lifetime combination medications vs all other combinations and is unadjusted. We could not adjust for it because of the small sample size. OR = odds ratio.

## Discussion

This retrospective study of youth who were prescribed stimulant and nonstimulant medications for ADHD found a low risk of newly diagnosed psychotic symptoms in 2.6% of cohort members. Exposure to amphetamines and to atomoxetine was associated with increased risk of newly diagnosed psychotic symptoms compared with nonexposure to each study medication, after adjusting for age at diagnosis of ADHD and sex assigned at birth. Additional analyses showed that the association between atomoxetine and newly diagnosed psychotic symptoms was higher among cohort members with exposure to atomoxetine combined stimulants (amphetamines, methylphenidate, or both) compared with those with exposure to atomoxetine monotherapy.

Our results align with previously published findings that document an association between exposure to amphetamines and increased risk for psychosis.^22^^,^^23^ Our findings are based on an observational retrospective cohort study design; therefore, a causal link between amphetamine exposure and newly diagnosed psychotic symptoms was not demonstrated. That said, numerous studies have shown that amphetamines, which increase synaptic levels of dopamine, can exacerbate primary psychotic symptoms of schizophrenia and can induce schizophrenia-like psychotic symptoms.^24^ Of interest, while acute psychotic symptoms associated with stimulant use may be expected to resolve within days in otherwise healthy individuals, recovery can be incomplete for some.^5^^,^^24^ There is increasing evidence that a history of ADHD during childhood is associated with increased risk of developing a psychotic disorder, including schizophrenia.^24^

Patients with ADHD present with comorbidities, most commonly substance use disorders, mood and anxiety disorders, and psychotic disorders,^25^^,^^26^ which may have affected the response to medication and risk of developing newly diagnosed psychotic symptoms. As such, several studies on the risk of recurrent or severe psychotic symptoms with use of amphetamines focused on participants with chronic stimulant abuse. Our study investigated such effects in youth receiving amphetamines and other medications for ADHD, presumably without abuse. However, these findings have relevance to our current study, given the high rates of substance use disorder among patients with ADHD^27^ and the inability to control for abuse of stimulant prescriptions. On the other hand, a study was recently published that found no evidence between stimulant treatment and later development of substance use disorder,^28^ and successful treatment of ADHD with amphetamines and other agents has been associated with lower risk for substance use disorders than untreated ADHD.^19^ Our findings warrant further investigation of the role of ADHD comorbidities in the development of psychotic symptoms.

The age of our cohort members poses an important consideration: the presentation of attentional difficulties as prodromal symptoms of schizophrenia. Youth at risk of schizophrenia present initially with problems in cognition, information processing, and attention difficulties.^29^, ^30^, ^31^ These cognitive difficulties could have been misinterpreted as ADHD and treated with psychostimulants. Additionally, youth with prodromal symptoms are more likely to receive various trials of ADHD medications in efforts to address their cognitive and attentional difficulties. Such groups of youth are likely at higher risk of developing psychotic symptoms, and we suspect that treatment with stimulants may increase that risk. However, we merely report an association that warrants further investigation. Our findings highlight the need for additional studies to determine if psychotic symptoms with ADHD pharmacotherapy are part of a prodromal presentation of schizophrenia, or from medication use, or a combination of both (ADHD pharmacotherapy increasing the risk of psychosis in subgroups with prodromal symptoms or family history of psychosis). Our findings highlight that further studies are required to identify risk factors, such as family history of psychosis, for severe or persisting psychotic symptoms associated with ADHD treatment using stimulants, amphetamines in particular, in youth.

Amphetamine exposure, but not methylphenidate exposure, was associated with newly diagnosed psychotic symptoms. Our findings were consistent with a large retrospective cohort study that documented a significantly higher risk of new-onset psychotic symptoms with amphetamines than methylphenidate in a large cohort of patients (13-25 years of age) with diagnosed ADHD.^15^ In the context of our findings that replicate a previous large-scale epidemiological study^15^ and the abundance of evidence that amphetamines increase dopamine more than methylphenidate,^5^^,^^9^ a plausible explanation for our findings is that psychotic symptoms emerge as a more common side effect of amphetamines in youth with ADHD. However, this remains unclear and warrants a large-scale randomized clinical trial to test this finding.

Differences in the pharmacological effects of amphetamines and methylphenidate may also be relevant to the risk of newly diagnosed psychotic symptoms. For example, both amphetamines and methylphenidate inhibit dopamine and norepinephrine transporters; however, preclinical studies have shown that dextroamphetamine results in substantially larger increases in synaptic and extracellular dopamine than methylphenidate at clinically relevant doses,^22^ an effect that may be related to the property of amphetamines in inducing the direct release of dopamine beyond its effects on the dopamine transporter.^32^

This study provides novel data on atomoxetine and the risk of newly diagnosed psychotic symptoms. Atomoxetine is a norepinephrine reuptake inhibitor that has been shown in preclinical models to increase extracellular levels of dopamine in prefrontal cortex.^33^^,^^34^ However, in contrast to stimulants, atomoxetine has not been consistently shown to increase striatal dopamine.^33^^,^^34^ Even though the prescribing information for atomoxetine includes a warning related to the emergence of new psychotic or manic symptoms, cases of psychosis following exposure to atomoxetine are rarely reported, and in cases where psychosis was reported, it was most often associated with comorbid psychiatric conditions such as mood and anxiety disorders.^35^ The association between atomoxetine exposure and increased risk of newly diagnosed psychotic symptoms observed in this study is consistent with a report of 360 cases of atomoxetine-induced psychosis or mania that were captured in the drug manufacturers’ databases between 2000 and 2005, although 110 of these cases were not clinically confirmed.^14^ A small registry-based study reported 4 cases of transient psychosis out of 96 (4.2%) pediatric patients taking atomoxetine for ADHD.^36^ None of these databases reported on confounding factors such as prodromal symptoms or family history of psychosis. These findings are contradictory, however, to findings from most clinical studies documenting no such associations between atomoxetine and newly diagnosed or treatment-emergent psychotic symptoms.^34^^,^^36^, ^37^, ^38^, ^39^, ^40^ Importantly, associations between atomoxetine and newly diagnosed psychotic symptoms in this study were significant only when cohort members were also prescribed stimulants. The atomoxetine/stimulant lifetime combination may suggest that a cohort of youth with ADHD did not respond to regular treatment, which led to polypharmacy. In such cohorts of youth, ADHD that is deemed resistant to treatment may indeed be a risk factor for subsequent development of psychotic symptoms.

Channeling bias is another potential explanation of the observed association between atomoxetine and newly diagnosed psychotic symptoms in this study, given that atomoxetine would be preferred over stimulants in youth with ADHD who are presumed to be at higher risk of a primary psychotic illness (for example, based on a personal or family history of psychotic spectrum illness or presentation of prodromal symptoms) or mania (based on a personal or family history of bipolar spectrum illness). Channeling bias is a less satisfactory explanation for our study findings with atomoxetine, however, given no significant association between newly diagnosed psychotic symptoms and α-2 agonists. Similar to atomoxetine, α-2 agonists appear to be essentially devoid of pro-dopaminergic effects in areas of the brain that have been implicated in the risk of psychosis or psychotic symptoms. That said, it is not possible to conclude that methylphenidate and α-2 agonists alone do not increase the risk of psychotic symptoms based only on the results of this study.

Interestingly, we found an increased risk of newly diagnosed psychotic symptoms in male youth treated for ADHD compared with female youth. There has been limited investigation of potential sex differences in the risk of newly diagnosed psychotic symptoms among youth who are treated with pharmacotherapeutic agents for ADHD. A study from Taiwan with a large population found that female sex in pediatric patients with ADHD was significantly associated with psychotic disorders in general and schizophrenia in particular.^35^ However, the study did not report on the interaction between sex and medication exposure. Additional research on a potential sex dimorphism regarding the risk of newly diagnosed psychotic symptoms with ADHD pharmacotherapy, stimulants in particular, is needed.

Strengths of this study include using a US population–based, geographically defined cohort to study the risk of newly diagnosed psychotic symptoms associated with approved ADHD treatments in youth. The methodological approach in this study accounted for the complex temporal nature of medication prescription with ADHD management by using time-varying covariate Cox proportional hazard regression models. Our study is one of the few studies to examine the risk of newly diagnosed psychotic symptoms in youth with ADHD that focused on associations with a full spectrum of approved medications for that indication.

The overall risk of psychotic symptoms in the study population is small. Therefore, the comparisons in this study were based on only a small number of events of psychotic symptoms. We did not have information on ethnicity, which limited our ability to find direct implications of our findings based in diverse populations. We did not have complete information on study drug doses or duration of exposures to study medications for all cohort members, which limited our ability to provide dose-response relationships between study drugs and newly diagnosed psychotic symptoms or associations by cumulative exposures.

This study did not include a referent group of youth not receiving pharmacotherapy for ADHD (nonuser control group) or a referent group of youth without attentional difficulties. This study did not have scales to assess for prodromal symptoms and baseline cognitive function; this limited our ability to infer if psychotic symptoms are best explained as prodromal symptoms or secondary to ADHD pharmacotherapy. The use of other medications was not assessed, impacting the external validity of our study. It was not possible to exclude all other medications, as this would have had adverse effects on the power of our study. Given that our study interest was the association between ADHD medications and newly diagnosed psychotic symptoms, we excluded medications that could mimic psychotic symptoms and cohort members with substance use disorders. Our approach of restricting our cohort based on exposure to medications that could lead to psychotic symptoms was similar to previously published studies.

The REP database has rich information, but its small size compared with national population-based registries presents with some challenges. Excluding other ADHD comorbidities, such as anxiety disorders, was not feasible in our study because it resulted in a low-powered study. However, comparisons between ADHD medication users and nonuser controls may be expected to be highly subject to bias (given expected differences in the risk of psychotic symptoms and other severe adverse effects of ADHD medications) and less clinically valid (given that pharmacotherapy is the predominant form of treatment for the majority of youth with ADHD). The data for the study were derived from electronic records through the REP that were not collected for research purposes and are therefore subject to misclassification and lacking important clinical information on confounding factors such as pretreatment ADHD severity, provider prescribing habits, undiagnosed bipolar spectrum illness or anxiety or mood disorders, and family history of psychosis. As such, we were not able to identify specific codes for early-onset psychotic symptoms or use other diagnostic tools such as *DSM*. The process of manual review of the electronic records to validate diagnoses and confounding factors was not possible for the available resources for this study and would be a consideration for a future follow-up study.

We were able to analyze the study drugs over a lifetime only and not over longitudinal time, which is a limitation of a retrospective cohort design. The atomoxetine/stimulant combination was based on lifetime combinations, which limited our ability to draw conclusions based on co-exposure of both medication classes. A prospective study design would have made such analysis possible overcoming this limitation but was not logistically feasible given the size of the sample required to observe meaningful differences in risk between exposures and the associated cost. However, a retrospective cohort study, such as this one, allows the study of rare or uncommon outcomes of treatment, such as newly diagnosed psychotic symptoms. Finally, in this study, we were unable to compare the longitudinal course of treatment and untreated ADHD as it pertains to the risk of newly diagnosed psychotic symptoms.

In conclusion, the absolute risk of psychotic symptoms was low overall. Treatment with amphetamines was associated with increased risk of newly diagnosed psychotic symptoms in a cohort of youth with diagnosed ADHD. An association between atomoxetine and an increased risk of newly diagnosed psychotic symptoms was observed, but was likely driven by individuals exposed to lifetime combinations of atomoxetine and stimulants. This study provides novel findings in the pediatric ADHD population on potential risks of newly diagnosed psychotic symptoms associated with exposure to the full spectrum of approved medications for the treatment of ADHD in youth. This study highlights the importance of further studies identifying risk factors, such as family history of psychosis and prodromal symptoms of psychosis, when treating a cohort of youth with amphetamine or atomoxetine.
