# Supplementary material for: Risk of Newly Diagnosed Psychotic Symptoms in Youth Receiving Medications for Attention-Deficit/Hyperactivity Disorder
Source: JAACAP Open. 2024 Feb 5;2(2):135–44. doi: 10.1016/j.jaacop.2024.01.003 (PMC11562438; doi:10.1016/j.jaacop.2024.01.003)
Supplement: Supplemental Table 1 [file mmc1.docx]

**Table S1: Diagnosis List and Diagnostic ICD code**

| **DIAGNOSIS**  **ICD-CODE** | **DSM-5 DIAGNOSIS LIST** |
| --- | --- |
| **292.12** | DRUG-INDUCED HALLUCINOSIS |
| **292.84** | DRUG-INDUCED ORGANIC AFFECTIVE SYNDROME |
| **292.89** | OTHER SPECIFIED DRUG-INDUCED MENTAL DISORDERS |
| **292.9** | UNSPECIFIED DRUG-INDUCED MENTAL DISORDER |
| **294.9** | UNSPECIFIED PERSISTENT MENTAL DISORDERS DUE TO CONDITIONS CLASSIFIED ELSEWHERE |
| **295.00** | SIMPLE TYPE SCHIZOPHRENIA, UNSPECIFIED STATE |
| **295.02** | SIMPLE TYPE SCHIZOPHRENIA, CHRONIC STATE |
| **295.10** | DISORGANIZED TYPE SCHIZOPHRENIA, UNSPECIFIED STATE |
| **295.30** | PARANOID TYPE SCHIZOPHRENIA, UNSPECIFIED STATE |
| **295.32** | PARANOID TYPE SCHIZOPHRENIA, CHRONIC STATE |
| **295.34** | PARANOID TYPE SCHIZOPHRENIA, CHRONIC STATE WITH ACUTE EXACERBATION |
| **295.40** | ACUTE SCHIZOPHRENIC EPISODE, UNSPECIFIED STATE |
| **295.45** | ACUTE SCHIZOPHRENIC EPISODE, IN REMISSION |
| **295.70** | SCHIZO-AFFECTIVE TYPE SCHIZOPHRENIA, UNSPECIFIED STATE |
| **295.72** | SCHIZO-AFFECTIVE TYPE SCHIZOPHRENIA, CHRONIC STATE |
| **295.80** | OTHER SPECIFIED TYPES OF SCHIZOPHRENIA, UNSPECIFIED STATE |
| **295.82** | OTHER SPECIFIED TYPES OF SCHIZOPHRENIA, CHRONIC STATE |
| **295.90** | UNSPECIFIED TYPE SCHIZOPHRENIA, UNSPECIFIED STATE |
| **295.92** | UNSPECIFIED TYPE SCHIZOPHRENIA, CHRONIC STATE |
| **296.24** | MAJOR DEPRESSIVE AFFECTIVE DISORDER, SINGLE EPISODE, SEVERE DEGREE, SPECIFIED AS WITH PSYCHOTIC BEHAVIOR |
| **296.34** | MAJOR DEPRESSIVE AFFECTIVE DISORDER, RECURRENT EPISODE, SEVERE DEGREE, SPECIFIED AS WITH PSYCHOTIC BEHAVIOR |
| **296.44** | BIPOLAR AFFECTIVE DISORDER, MANIC, SEVERE DEGREE, SPECIFIED AS WITH PSYCHOTIC BEHAVIOR |
| **296.54** | BIPOLAR AFFECTIVE DISORDER, DEPRESSED, SEVERE DEGREE, SPECIFIED AS WITH PSYCHOTIC BEHAVIOR |
| **296.64** | BIPOLAR AFFECTIVE DISORDER, MIXED, SEVERE DEGREE, SPECIFIED AS WITH PSYCHOTIC BEHAVIOR |
| **296.80** | MANIC-DEPRESSIVE PSYCHOSIS, UNSPECIFIED |
| **296.89** | OTHER MANIC-DEPRESSIVE PSYCHOSIS |
| **296.90** | UNSPECIFIED AFFECTIVE PSYCHOSIS |
| **296.99** | OTHER SPECIFIED AFFECTIVE PSYCHOSES |
| **297.1** | PARANOIA |
| **297.9** | UNSPECIFIED PARANOID STATE |
| **298.1** | EXCITATIVE TYPE PSYCHOSIS |
| **298.8** | OTHER AND UNSPECIFIED REACTIVE PSYCHOSIS |
| **298.9** | UNSPECIFIED PSYCHOSIS |
| **299.80** | OTHER SPECIFIED EARLY CHILDHOOD PSYCHOSES, CURRENT OR ACTIVE STATE |
| **299.81** | OTHER SPECIFIED EARLY CHILDHOOD PSYCHOSES, RESIDUAL STATE |
| **299.90** | UNSPECIFIED CHILDHOOD PSYCHOSIS, CURRENT OR ACTIVE STATE |
| **299.91** | UNSPECIFIED CHILDHOOD PSYCHOSIS, RESIDUAL STATE |
| **F23** | BRIEF PSYCHOTIC DISORDER |
| **F23** | Brief psychotic disorder |
| **F24** | Shared psychotic disorder |
| **F25.0** | SCHIZOAFFECTIVE DISORDER, BIPOLAR TYPE |
| **F25.0** | Schizoaffective disorder, bipolar type |
| **F25.1** | SCHIZOAFFECTIVE DISORDER, DEPRESSIVE TYPE |
| **F25.1** | Schizoaffective disorder, depressive type |
| **F25.8** | Other schizoaffective disorders |
| **F25.9** | SCHIZOAFFECTIVE DISORDER, UNSPECIFIED |
| **F25.9** | Schizoaffective disorder, unspecified |
| **F28** | OTHER PSYCHOTIC DISORDER NOT DUE TO A SUBSTANCE OR KNOWN PHYSIOLOGICAL CONDITION |
| **F28** | Other psychotic disorder not due to a substance or known physiological condition |
| **F29** | UNSPECIFIED PSYCHOSIS NOT DUE TO A SUBSTANCE OR KNOWN PHYSIOLOGICAL CONDITION |
| **F29** | Unspecified psychosis not due to a substance or known physiological condition |

Note: DSM-V = Diagnostic Statistical Manual, 5^th^ edition
